# Azole-Resistant COVID-19-Associated Pulmonary Aspergillosis in an Immunocompetent Host: A Case Report

**DOI:** 10.3390/jof6020079

**Published:** 2020-06-06

**Authors:** Eelco F. J. Meijer, Anton S. M. Dofferhoff, Oscar Hoiting, Jochem B. Buil, Jacques F. Meis

**Affiliations:** 1 Department of Medical Microbiology, Radboud University Medical Center, 6500HB Nijmegen, The Netherlands; Eelco.Meijer@radboudumc.nl (E.F.J.M.); Jochem.Buil@radboudumc.nl (J.B.B.); 2 Center of Expertise in Mycology Radboudumc/CWZ, 6532 SZ Nijmegen, The Netherlands; 3 Department of Medical Microbiology and Infectious Diseases, Canisius Wilhelmina Hospital (CWZ), 6532 SZ Nijmegen, The Netherlands; A.Dofferhoff@cwz.nl; 4 Department of Internal Medicine, Canisius Wilhelmina Hospital (CWZ), 6532 SZ Nijmegen, The Netherlands; 5 Department of Intensive Care Medicine, Canisius Wilhelmina Hospital (CWZ), 6532 SZ Nijmegen, The Netherlands; O.Hoiting@cwz.nl; 6 Bioprocess Engineering and Biotechnology Graduate Program, Federal University of Paraná, Curitiba 81531-970, PR, Brazil

**Keywords:** SARS-CoV-2, co-infection, pulmonary aspergillosis, ICU, azole-resistant *Aspergillus*, *Aspergillus fumigatus*, CAPA, TR_34_L98H

## Abstract

COVID-19-associated pulmonary aspergillosis (CAPA) is a recently described disease entity affecting patients with severe pulmonary abnormalities treated in intensive care units. Delays in diagnosis contribute to a delayed start of antifungal therapy. In addition, the emergence of resistance to triazole antifungal agents puts emphasis on early surveillance for azole-resistant *Aspergillus* species. We present a patient with putative CAPA due to *Aspergillus fumigatus* with identification of a triazole-resistant isolate during therapy. We underline the challenges faced in the management of these cases, the importance of early diagnosis and need for surveillance given the emergence of triazole resistance.

## 1. Introduction

There have been suggestions that coronavirus disease 2019 (COVID-19) might increase the risk of superinfections [1] and, particularly, invasive pulmonary aspergillosis (IPA) co-infection [2]. COVID-19-associated pulmonary aspergillosis (CAPA) is a recently described disease entity affecting patients in intensive care unit (ICUs) with severe pulmonary abnormalities. Small cohorts of 31 patients in the Netherlands [3], 27 patients in France [4] and 19 patients in Germany [5] have been published, showing CAPA rates of 19.4%, 33% and 26%, respectively. An additional two fatal cases of CAPA were recently reported [6,7]. The numbers resemble what has been observed in influenza, where influenza in ICU patients has been identified as an independent risk factor for invasive pulmonary aspergillosis and which is associated with an even higher mortality rate than IPA alone [8]. In addition, in the Netherlands, an estimated 11.3% of cases with invasive aspergillosis are infected with an azole-resistant isolate [9], potentially increasing mortality to 50–100% [10]. We present the first case of azole-resistant *Aspergillus fumigatus* in a SARS-CoV-2-positive immunocompetent patient admitted to the ICU.

## 2. Case Report and Results

A 74-year-old patient was admitted because of respiratory insufficiency amid the COVID-19 crisis. Eleven days prior to admission, she had been suffering from fever (38.5 °C) and a dry cough. Three days after symptom onset, she developed diarrhea. Her medical history included complaints of reflux and pain due to arthrosis of the hip and knees, for which she uses a proton-pump inhibitor and a nonsteroidal anti-inflammatory drug pantoprazol and etoricoxib, respectively. She stopped smoking 20 years ago and was healthy and fit otherwise. Patient characteristics can be found in Table 1. This study, “Clinical course and prognostic factors for COVID-19” with project identification code CWZ-nr 027-2020, was approved in March 20202 by the Canisius Wilhelmina Hospital medical ethics committee and patient informed consent was acquired antemortem with opt out possibility.

**Figure 1:**
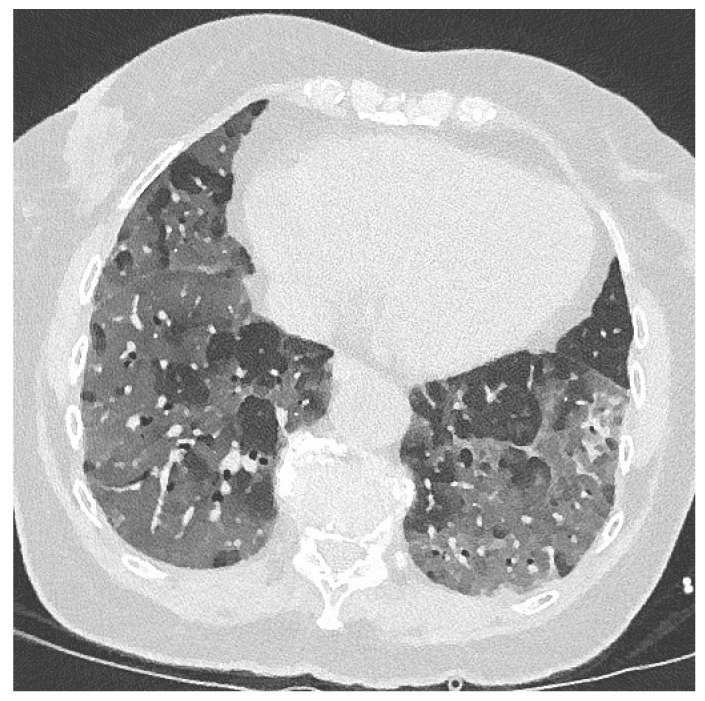
Low-dose chest CT showing extensive centralized and peripheral bilateral ground glass opacities with left-sided consolidations and bilateral fibrotic bands. No pleural effusion. No vascular enlargement and no specific suggestions of aspergillosis.

At presentation to the emergency department, she had been feeling progressively dyspneic for two days. On physical examination, her oxygenation was 82%, with 28 breaths per minute in room air, pulmonary wheezing and an extended expiration. Oxygenation improved to 94% with 5 L O_2_ via a nasal cannula, but she desaturated during speech. Her BMI was 27.7 (80 kg) and her temperature 37.8 °C. No other aberrant observations on physical examination were made. Her Glasgow Coma Scale was 15 and her ECG was normal. Her C-reactive protein (CRP) was 214 mg/L, and other laboratory findings included slightly elevated leucocytes (12.6 × 10^9^ /L) and neutrophils (8.4 × 10^9^ /L), elevated liver enzymes (alkaline phosphatase 528 U/L; GGT 376 U/L; AST 76 U/L; LD 745 U/L), slightly elevated pro-calcitonin (0.25 µg/L; <0.5 µg/L not suggestive of bacterial infection), increased ferritin (1442 µg/L), and normal electrolyte, glucose and renal function. SARS-CoV-2 nasopharyngeal and throat swabs were taken. A low-dose chest CT demonstrated extensive centralized and peripheral bilateral ground glass opacities with left-sided consolidations and bilateral fibrotic bands without pleural effusions and vascular enlargement **(**Figure 1). The CO-RADS score was 5 and CT-severity score was 24 out of 25 [11]. 

**Figure 2:**
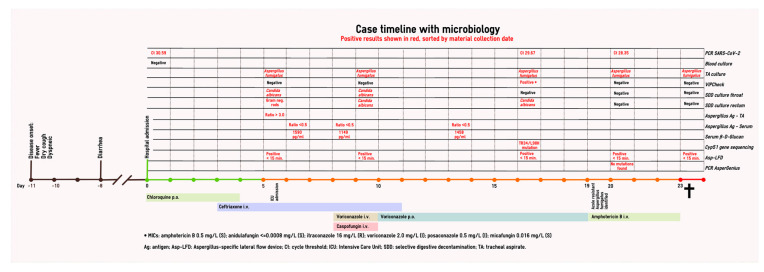
Case timeline with microbiology.

Because of the high probability of SARS-CoV-2 infection, chloroquine treatment was started (600 mg and 300 mg on day 1, 300 mg q12h days 2–5), which was national policy at the time. The SARS-CoV-2 PCR of a nasopharyngeal swab was positive (Ct 30.59; E gene [12]). Blood cultures remained negative, as were nasopharynx bacterial cultures taken at admission. The patient was subsequently admitted to our general inpatient respiratory ward. An overview of her hospital course is depicted in Figure 2.

The CRP remained highly stable over the following days at around 200 mg/L with a range of 192–214. However, the patient needed increasing oxygenation with a non-rebreathing mask. Empirical treatment of a suspected bacterial superinfection was started with ceftriaxone i.v. 2000 mg q24h. Five days after admission, the maximum (15 L O_2_) oxygenation with the non-rebreathing mask became insufficient and the patient was admitted to the ICU for respiratory support and intensive monitoring.

In the ICU, HFNO (high-flow nasal oxygen therapy) and selective digestive decontamination (SDD) were initiated, which includes ceftriaxone i.v. 2000 mg q24h for 4 days and a combined oral non-absorbable suspension of amphotericin B, colistin and tobramycin q6h. In this patient, ceftriaxone was continued de facto for another 4 days. Routine bacterial and fungal (peri-anal, throat and tracheal aspirate) surveillance cultures were done twice weekly in adherence with our local SDD policy [13]. Within a few hours after admission to the ICU, her blood oxygenation became insufficient with HFNO at FiO2 100% and 60 L/min flow. Therefore, she was sedated, intubated and put on a mechanical ventilator. A CT angiography of the chest was performed which demonstrated significant bilateral pulmonary emboli. Anticoagulants (enoxaparine anti-factor Xa) were initiated in therapeutic dosages. Pressure control ventilation was required with the patient in prone position. Because of the need for increasing noradrenaline dosages during circulatory shock, hydrocortisone 100 mg q8h was initiated and continued for five days. Cardiac ultrasound showed a minor tricuspid insufficiency but no major pathology. 

*Aspergillus fumigatus* was recovered from high-volume tracheal aspirate cultures [14] obtained at ICU admission. Aspergillus galactomannan (Platelia Aspergillus; Bio-Rad, Marnes-La-Coquette, France) ratio at this time was >3.0 (positive) in a tracheal aspirate and β-d-glucan (Fungitell assay; Associates of Cape Cod Inc., East Falmouth, MA, USA) in serum was 1590 pg/mL (positive), after which a putative diagnosis of CAPA was made. Serum galactomannan remained negative (<0.5) in three subsequent samples. Voriconazole i.v. 6 mg/kg q12h was started in addition to caspofungin i.v. 70 mg q24h until the VIPcheck (Mediaproducts BV, Groningen, The Netherlands), used to detect azole resistance, was negative. MICs determined with broth microdilution using CLSI methodology of the *A. fumigatus* isolate were as follows: amphotericin B 0.5 mg/L, micafungin and anidulafungin <0.016 mg/L, itraconazole 1 mg/L, voriconazole 0.25 mg/L, and posaconazole 0.063 mg/L. Voriconazole was switched to oral administration of 200 mg q12h with discontinuation of caspofungin. During SDD, bacterial cultures remained negative throughout her stay in the ICU. 

On day 6 after admission (day 2 at the ICU), continuous venovenous hemofiltration was initiated because of rapidly progressive acute renal failure. *A. fumigatus* was persistently cultured from tracheal aspirate samples during voriconazole treatment and β-d-glucan levels remained positive with 1149 and 1458 pg/µl, at 1 and 6 days (day 8 and 13 after hospital admission) of voriconazole therapy, respectively. Voriconazole serum therapeutic drug monitoring was performed as recommended [15], with therapeutic concentrations of 4.72 mg/L, 2.78 mg/L and 1.43 mg/L at day 13, 15 and 17, respectively.

The respiratory situation improved marginally in the subsequent 7 days but declined steadily thereafter. Pressure support and pressure control ventilation were alternated between days 12 and 19 and attempts to return the patient to a supine position failed several times. After 7 days, *A. fumigatus* grew on the itraconazole and voriconazole wells of the second VIPcheck on day 19 (tracheal aspirate culture). MICs of this *A. fumigatus* isolate were as follows: amphotericin B 0.5 mg/L, anidulafungin and micafungin <0.016 mg/L, itraconazole 16 mg/L, voriconazole 2 mg/L and posaconazole 0.5 mg/L. Voriconazole treatment was changed to liposomal amphotericin B 200 mg q24h. Subsequent *cyp51A* gene sequencing identified a TR_34_/L98H mutation, probably responsible for the observed azole resistance. On day 22, ventilation and oxygenation of the patient deteriorated further without further treatment options and therapy was discontinued on day 23. An autopsy was not performed.

## 3. Discussion

We report the first case of azole-resistant CAPA, which occurred in an immunocompetent host during ICU support without a previous history of azole therapy. The *A. fumigatus cyp51A* gene TR_34_/L98H mutation found in this patient has been well described as an environmentally acquired mutation [16], which is in line with data from clinical studies where two-thirds of patients with azole-resistant infections had no history of azole pretreatment [10]. This case underscores the importance of early diagnosis and the need for resistance surveillance, comparable to what has been described in influenza patients [9,17], given the emergence of triazole resistance [18,19].

The sensitivity for detection of resistance in primary cultures with the VIPcheck plate depends on the number of *A. fumigatus* colonies that are tested, as clinical cultures may contain both mixed azole-susceptible and azole-resistant isolates during an infection [20]. We suspect that *A. fumigatus* isolated in the first tracheal aspirate was already a mixed culture but was missed in initial fungal cultures due to abundance of azole-susceptible *A. fumigatus* spores. Molecular detection could have given a suggestion to the presence of a mixed culture [21] but PCR could not be performed due to absence of material. The TR_34_/L98H had a phenotype with high itraconazole MIC (>16 mg/L) and low voriconazole MIC (2 mg/L), similar to strains which have been described only recently in the Netherlands [22].

IPA is known to be problematic to diagnose in the non-neutropenic ICU host [23]. Regardless of the compelling evidence for CAPA in this patient, the EORTC/MSGERC [24] host criteria for invasive fungal disease were not met, nor did the patient meet the AspICU algorithm because we tested tracheal aspirates instead of bronchoalveolar lavage (BAL) fluid [25]. This is in line with findings from other groups, where CAPA patients did not meet the EORTC/MSGERC host criteria either [3,4,5,6]. In addition, the American Association for Bronchology and Interventional Pulmonology (AABIP) has issued a statement advising against routine bronchoscopy in COVID-19 patients, as it poses substantial risk to patients and staff [26]. BAL should only be considered in intubated patients if upper respiratory samples are negative and BAL would significantly change clinical management. Tracheal aspirate cultures, as performed twice weekly in our patient, repeatedly identified *A. fumigatus* as the only micro-organism present. In the first positive culture, five colonies were tested for resistance with the VIPcheck plate as is recommended to exclude azole resistance [15]. When surveillance cultures of tracheal aspirates were persistently cultured positive with *A. fumigatus* during voriconazole therapy, we suspected the selection of resistant isolates which were probably already present in the first samples, albeit in undetectable numbers. An autopsy to confirm IPA was not done.

Serum galactomannan testing has been shown to be a fairly sensitive diagnostic tool (70%) in neutropenic patients with pathology-proven invasive aspergillosis [27,28]. However, in patients who are non-neutropenic, serum galactomannan sensitivity of around 25% has been reported [27], which may explain the low number of serum galactomannan positive findings in recently published case reports [6,7] and case series [3,4,5]. The role of β-d-glucan and the *Aspergillus*-specific lateral flow device (LFD) as an adjunct to the diagnosis of IPA in COVID-19 is not yet clear [2,23]. Serum β-d-glucan was persistently strongly positive in this patient over the course of a week. The specificity for invasive fungal disease of β-d-glucan testing in a mixed ICU population has been shown to be high (86%), with two consecutive positive results [29] compared to those with only fungal colonization and no invasive fungal disease. In addition, multiple other studies report a good sensitivity for the diagnosis of invasive aspergillosis in critically ill patients [30,31,32,33,34]. BAL β-d-glucan in the ICU setting is, however, not recommended, due to its poor specificity and confounders causing false positive results [35].

The LFD is particularly interesting in the ICU due to its short turnaround time. It has demonstrated a higher sensitivity but lower specificity in BAL fluids compared to galactomannan [36] and β-d-glucan [37] in IPA-probable and proven immunocompromised patients. In the ICU setting, however, LFD is suggested to have a lower sensitivity but comparable specificity to galactomannan testing in BAL fluids [35,38]. Noteworthily, a negative predictive value of >96% has been reported in the ICU setting [39]. We used the OLM lateral flow device (AspLFD) on sequential patient tracheal aspirates, yielding positive results on all samples confirming the positive galactomannan result. Although suitable for its negative predictive value or as an additional diagnostic measure, further evaluation of lateral flow technology in critically ill patients is warranted.

Altogether, we describe the clinical course of the first reported patient with azole-resistant CAPA. The contribution of *A. fumigatus* to this fatal COVID-19 course is highly likely, although autopsy was not performed, as in all previously reported CAPA cases [3,4,5,6,7].

## Figures and Tables

**Table 1:** Patient characteristics

Gender		Female
Age (years)		74
Medical history		Reflux, polyarthrosis, stopped smoking 20 years ago
Medication		Pantoprazol (PPI) and Etoricoxib (NSAID)
Underlying immuno-compromising condition		None
Initial symptoms		Fever, dry cough, dyspneic, diarrhea
ARDS	Prone positioning	Yes
	vvECMO	No
Acute renal failure		Yes, continuous venovenous hemofiltration (CVVH)
IPA definition	EORTC/MSG criteria	N/A
	(modified) AspICU algorithm	N/A
